# Draft Genome Sequence of a Bombella apis Strain Isolated from Honey Bees

**DOI:** 10.1128/MRA.01329-19

**Published:** 2019-11-21

**Authors:** Eric A. Smith, Sylvie A. Martin-Eberhardt, Delaney L. Miller, Audrey J. Parish, Irene L. G. Newton

**Affiliations:** a Department of Biology, Indiana University, Bloomington, Indiana, USA; University of Maryland School of Medicine

## Abstract

Bombella apis occupies a variety of distinct niches within a honey bee hive, including queen guts, royal jelly, and larval food. In an effort to better understand its evolution and identify signatures of honey bee association, we sequenced a strain isolated from hive honey stores. This genome is 2,086,308 bp long and contains 1,975 protein-coding genes.

## ANNOUNCEMENT

The honey bee (Apis mellifera) is extremely important economically because of the pollination services it provides to numerous agricultural crops. There has been increased interest of late in the role of the microbiome in honey bee health. These studies have identified a core worker microbiome of 8 to 10 bacterial species (1–6). However, less interest has been given to the honey bee queen microbiome. The recently described Bombella apis (7) has been found to occupy a variety of niches within the hive, including queen guts, royal jelly, worker jelly, food stores, and larval guts (8–12).

We isolated one strain of *B. apis* (SME1) from honey within our hives in Bloomington, Indiana, in May 2018. Initial samples were streaked onto MRS agar plates, after which single colonies were picked and grown in liquid culture. *B. apis* SME1 was grown in MRS medium at 34°C overnight, with aeration. Total DNA was extracted using the DNeasy blood and tissue kit (Qiagen, Hilden, Germany), and sequencing library preparation was performed using the NEBNext Ultra II DNA library preparation kit (NEB, Ipswich, MA, USA) according to the manufacturer’s protocols. The resulting library was subjected to 250-bp paired-end sequencing on the Illumina MiSeq platform (version 2 chemistry) at the Indiana University Center for Genomics and Bioinformatics (Bloomington, IN), generating 408,281 read pairs.

Initial *de novo* assembly of this strain was performed using MaSuRCA version 3.2.8 (13). Reads were not subjected to quality control (QC) prior to assembly, as MaSuRCA performs internal QC during the assembly process. Additionally, reads were randomly subsampled down to approximately 50× coverage prior to assembly. The completeness of the assembly was assessed using both BUSCO (14) and CheckM (15). Default parameters were used for all bioinformatics software noted above, unless otherwise mentioned.

The assembly resulted in 12 contigs comprising 2,086,308 bp and an *N*_50_ contig value of 455,874 bp. The GC content of *B. apis* SME1 is 59.56%. Annotation was carried out with Rapid Annotations using Subsystems Technology (RAST) (16), which predicted 1,975 protein-coding genes and 4 rRNAs.

### Data availability.

This whole-genome shotgun sequencing project has been deposited at DDBJ/ENA/GenBank under accession number WHNS00000000. The version described in this paper is version WHNS01000000. Sequencing reads have been deposited under BioSample accession number SAMN13042715.
